# Viability of *Toxoplasma gondii* tachyzoites in different conditions for parasite transportation

**DOI:** 10.14202/vetworld.2022.198-204

**Published:** 2022-01-29

**Authors:** Thi Thuy Nguyen, Ketsarin Kamyingkird, Waraphon Phimpraphai, Tawin Inpankaew

**Affiliations:** 1 Department of Parasitology, Faculty of Veterinary Medicine, Kasetsart University, Bangkok 10900, Thailand; 2 Department of Veterinary Medicine, Faculty of Animal Science and Veterinary Medicine, University of Agriculture and Forestry, Hue University, Hue, Vietnam; 3 Department of Veterinary Public Health, Faculty of Veterinary Medicine, Kasetsart University, Bangkok 10900, Thailand.

**Keywords:** preservation time, *T. gondii* tachyzoites, temperature, transportation, viability

## Abstract

**Background and Aim:**

*Toxoplasma gondii* tachyzoite is the infective stage that causes acute infection, leading to severe toxoplasmosis. The tachyzoite stage has been extensively used for several inoculation purposes, including antigen production, immunological studies, nutrition mechanisms, and *in vitro* drug trials. The use of fresh tachyzoites is required for inoculation in either *in vitro* or *in vivo* studies. However, there is a lack of information on preserving live tachyzoites during transportation from laboratories to inoculation sites. Therefore, this study aimed to validate suitable preservative conditions for maintaining live parasites by determining the survival and viability of *T. gondii* tachyzoites on the basis of different media, temperatures, and incubation times.

**Materials and Methods:**

The free live *T. gondii* tachyzoites were evaluated on their viability when maintained in different media without 5% Carbon dioxide (CO_2_). The purified tachyzoites of the RH and PLK strains were individually suspended in normal saline (NS), phosphate-buffered saline (PBS), minimum essential medium (MEM), and MEM with 10% fetal bovine serum (MEM-FBS) and incubated for 6 h at ice-cold (IC; 3-9°C) and room temperature (RT; 25°C). Parasite survival was measured at the 0, 1^st^, 2^nd^, 3^rd^, 4^th^, 5^th^, and 6^th^ h post-incubation using the trypan blue exclusion test.

**Results:**

The viability was in the range of 85.0%–91.0% for IC using NS and 81.0%–85.1% (IC) and 75.3%–77.5% (RT) using PBS. The viability was approximately 75.0%–83.0% (IC) and 70.0%–79.0% (RT) using MEM and MEM-FBS. There was a significant difference in the viability between the seven periods on the basis of one-way repeated Analysis of variance and Friedman analyses. Parasite survival slightly reduced (20.0%–30.0%) in NS and MEM-FBS at both temperatures during incubation. Notably, PBS could not support tachyzoite viability after 3 h post-incubation.

**Conclusion:**

NS was a suitable preservative for maintaining purified *T. gondii* tachyzoites during transportation at IC and RT without 5% CO_2_ supplementation. This could be a valuable medium for parasite transportation, especially when there is a large distance between the laboratory and inoculation site.

## Introduction

*Toxoplasma gondii*, an obligate intracellular protozoan, is a ubiquitous parasite that infects almost all warm-blooded animals, including humans. Most of the infected immunocompetent individuals develop either asymptomatic or mild clinical signs, whereas acute infection can cause encephalitis and retinochoroiditis, particularly in immunocompromised hosts, and congenital disease in seronegative pregnant women [1,2]. Furthermore, the proliferation and development of the tachyzoite stage induce a reduction in liver and brain cholesterol content and a decline in host immune responses, leading to acute infection [3].

During the *T. gondii* life cycle, three asexual stages can invade the cells, including sporozoite, which is only produced by sexual reproduction and released in the oocysts through felid feces; bradyzoite, a form of slow multiplication originating from the tissue cysts in chronic infection; and tachyzoite, a form of proliferation that is characteristic of the acute infection [4,5]. The tachyzoites can be maintained and produced in cell cultivation systems (*in vitro*) and animal models (*in vivo*), which makes it the most experimentally tractable organism among Apicomplexa and has been modeled in many studies [4]. Tachyzoites can be purified and used in several studies, including immunity responses, histopathology, and drug trials. Suspension of the purified tachyzoites in the appropriate media helps avoid unexpected responses of animal or host cells to the parasites [6-8]. To maintain high viability and survival rates for the parasites*, T. gondii* tachyzoites should be used for inoculation in an animal model [9,10] and in vitro study immediately after purification.

Heider *et al*. [9] and Räisänen [11] demonstrated that tachyzoites survive and remain infectious better in serum solutions than the free nutrient media, the viability of the parasites in phosphate-buffered saline (PBS) or normal saline (NS) was significantly high, compared to enriched media in other studies [12,13]. Besides, the sensitivity of *T. gondii* tachyzoites against low pH was found in an experiment in which simulated gastric fluid was used to examine the tachyzoites infectivity retention in different acidity [14]. In general, these studies revealed the sensitivity of purified tachyzoites to the extracellular environment; to keep them alive outside the host cells; the tachyzoites must be maintained in the cultivation media in a sealed container without Carbon dioxide (CO_2_) supplementation during transportation. Some experiments require long-distance and time-consuming transportation to carry the purified, live parasites to the experimental animal areas from the laboratory. Unfortunately, a standardized protocol or set of conditions for maintaining live, purified tachyzoites for long-distance transportation is unavailable. Therefore, appropriate media and temperatures to maintain the *T. gondii* purified tachyzoites’ viability and infectivity during long-distance transportation need to be identified.

The objective of this study was to validate the conditions for preserving live, purified *T. gondii* tachyzoites in different media at two different temperatures and incubation periods without a 5% CO_2_ supplement.

## Materials and Methods

### Ethical approval

This study was carried out under a project on toxoplasmosis in duck that adhered to strict guidelines of animal care and use under the Ethical Review Board of the Office of National Research Council of Thailand (NRCT) for the conduction of the scientific research. The Approval number of ACKU63-VET035 was granted by Kasetsart University’s Institution Animal Care and Use Committee. Besides, the biosafety number IBC-63-V04 was granted by Institutional Biosafety Committee, Faculty of Veterinary Medicine, Kasetsart University.

### Study period and location

The study was conducted from March to May 2020. The viability of *T. gondii* tachyzoites maintained in different conditions was measured in the laboratory at Department of Parasitology, Faculty of Veterinary Medicine, Kasetsart University.

### Preservative preparation

Four different media were used to preserve the *T. gondii* tachyzoites: PBS 1X (pH 7.4), NS 0.85%, with adjusted pH 7.4, minimum essential medium (MEM 1X with Earle’s salts and 2.0-mM L-glutamine [Gibco™, USA]), and MEM-fetal bovine serum (FBS) (MEM+10% FBS [Gibco]). PBS and NS were autoclaved at 121°C for 15 min at 1.1 bars using a Tomy SX-500 high-pressure steam sterilizer (U.P. Marketing General Supply Co. Ltd., Thailand). MEM and MEM-FBS were passed through 0.2-mm syringe filters (Millipore, United States). As MEM-FBS contains bovine serum albumin (BSA), it is not tailored for *in vivo* assay but for other biological experiments of *T. gondii*, so this medium was also investigated to compare with the other buffers. Freshly prepared preservatives were used in every experiment to prevent pH changes.

### *T. gondii* strains and parasite preparation

Tachyzoites of the *T. gondii* RH and PLK strains were used in this study. The parasites were maintained using vero cells in MEM cultivation media supplemented with 1% Pen Strep (Gibco), 0.1% anti-fungi (250-mg/mL, HyClone™, USA), and 5-8% FBS. Both *T. gondii* strains and vero cells were obtained from the Department of Protozoology, Faculty of Tropical Medicine, Mahidol University, Bangkok, Thailand. The *T. gondii* tachyzoites were cultivated every 4 days in vero cells. Harvesting of the tachyzoites was performed as described by Nguyen *et al*. [15]. The harvested tachyzoites were washed in cold media (PBS, NS, MEM, and MEM-FBS) using centrifugation 3 times at 448 g and 4°C for 10 min. The number of parasites was counted using a Neubauer chamber (0.1-mm depth, Blaubrand**^®^**, Germany), then diluted to 3×10^6^ tachyzoites/mL (3×10^3^ tachyzoites/μL) before transferring to the four different preservatives being tested. The parasites were preserved in 100 μL of each medium and maintained in 1.5-mL sterile tubes (Corning Life Science [Wujiang] Co., Ltd, China). The parasites were preserved at ice-cold (IC) conditions, where the temperature increased from 3°C in the 1^st^ h to 9°C in the 6^th^ h of incubation and room temperature (RT; 25°C).

### Trypan blue exclusion test

The viability of *T. gondii* tachyzoites was observed in triplicate using the trypan blue exclusion test as described by Strober [16]. An amount (100-μL) of 0.4% trypan blue (Gibco) was mixed well into each parasite suspension and incubated for 3 min at RT. The unstained and stained cells were considered live and dead tachyzoites, respectively. After staining, the parasites were counted within 3-5 min in a Neubauer chamber using the 40× objective lens of a light microscope (Olympus, Japan). Approximately 150 tachyzoites were counted in all subsquares of 1×1 mm in the central square of the chamber. The viability was calculated as the ratio of live tachyzoites per total tachyzoites count and expressed as the mean viability. The viability of tachyzoites was monitored at the 0, 1^st^, 2^nd^, 3^rd^, 4^th^, 5^th^, and 6^th^ hours post-incubation (hpi).

### Statistical analysis

Statistical analyses were conducted using the R software package version 3.6.3 (R Foundation for Statistical Computing, Vienna, Austria) [17]. Descriptive statistics, consisting of mean, median, standard error, the 25^th^ and 75^th^ quartiles, maximum and minimum of the viability of tachyzoites were reported. The normal distribution and homogeneity of variances were verified using the Shapiro test and Levene’s test, respectively. The Kruskal-Wallis test followed by Wilcoxon’s test was used to determine statistical significance between the viability of tachyzoites preserved in the four media. The *T. gondii* tachyzoite viability measured 7 times was analyzed using one-way repeated measures Analysis of variance (ANOVA) or the Friedman test, and *p<0.05, **p<0.01, and ***p<0.001 were tested to indicate significant differences.

## Results

### Effect of different media on the viability of *T. gondii* tachyzoites preserved at IC and RTs

**Figure-1:**
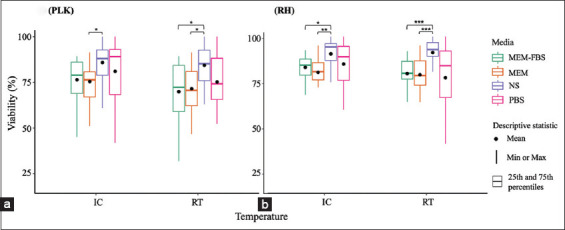
(a and b) Viability of *Toxoplasma gondii* tachyzoites preserved in four different media. Data of each medium at seven times are displayed as a box plot of the median (line), mean (dot), 25^th^ and 75^th^ percentiles (box), maximum and minimum (upper and whiskers, respectively).

The comparison of *T. gondii* tachyzoite viability in the different preservatives under the IC and RT conditions is shown in Figure-1 using the mean viability of parasites at the seven measurement periods. At RT, NS had the highest viability at 84.3% and 91.3% for the PLK and RH strains, respectively. The second highest viability in the PLK strain was 75.3% in PBS, yet this medium had the lowest proportion at 77.5% in the RH strain. In MEM and MEM-FBS, the viability of the PLK strain was about 70%, which was slightly lower than that of the RH strain (approximately 79%) (Figures-1a and b).

Similarly, at IC conditions, the highest viability was observed in NS with 85.8% and 90.6% for the PLK and RH strains, respectively, followed by viability in the PBS medium with 81.0% and 85.1% for the PLK and RH strains, respectively. For MEM-FBS, 76.4% of PLK tachyzoites and 83.2% of RH tachyzoites were confirmed viable during incubation. The viability of tachyzoites in MEM was 75.5% and 80.5% for the PLK and RH strains, respectively, which were the lowest proportions at this temperature (Figures-1a and b).

There was a significant difference between the mean viability of tachyzoites preserved using the four types of media for both temperature conditions as assessed using the Kruskal-Wallis test. Further examination on the basis of the Wilcoxon test showed that the mean viability was significantly higher in the NS medium than in the MEM-FBS and MEM, notably for the RH strain at IC (p<0.01) and RT (p<0.001) (Figure-1b).

### Viability of *T. gondii* tachyzoites during the preservation period

The viability of parasites in the different media was also monitored at different times. In general, the parasite’s survival decreased from 90-100% to 50-85% over the 6 h of incubation.

**Figure-2:**
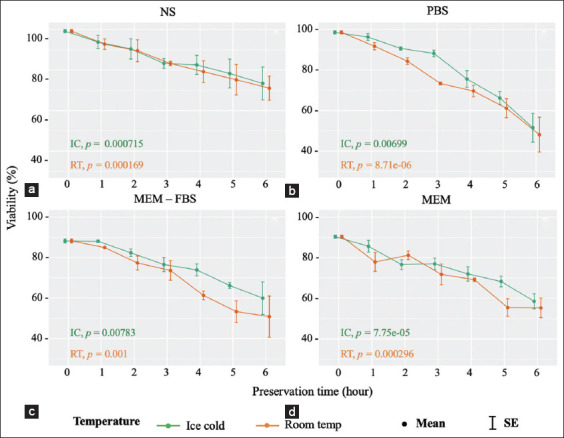
(a-d) Viability of *Toxoplasma gondii* PLK strain preserved in four different media (p indicates significant differences).

**Figure-3:**
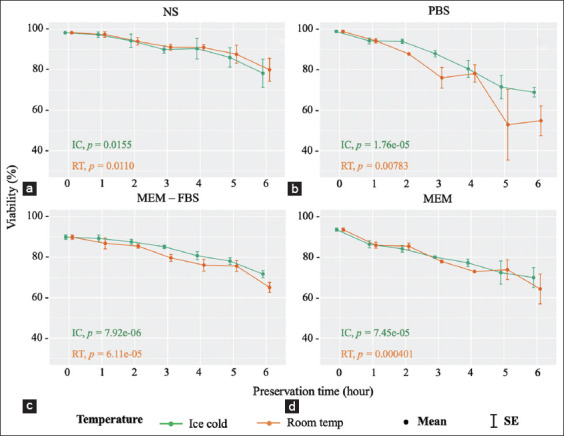
(a-d) Viability of *Toxoplasma gondii* RH strain preserved in four different media (p indicates significant differences).

There was no noticeable difference in the viability of parasites preserved using NS between the different temperatures. The viable rates of tachyzoites in this medium decreased steadily from just under 100% to over 70% (PLK strain) and <80% (RH strain) at the 6^th^ hpi (Figures-2a and 3a). At IC condition, PBS was only able to support viability at 3 hpi, but with a mild decline from about 100% to approximately 88% (both *T. gondii* strains), which was followed by substantial drops to 51.5% (PLK strain) and 68.8% (RH strain) in the past 3 h. At RT, the viability of tachyzoites in PBS medium fell moderately to 70% in both strains at the 3^rd^ hpi, then fluctuated and was lowest at 48.1% and 54.8% for PLK and RH strains, respectively, at the end of the period (Figures-2b and 3b).

With the MEM-FBS medium, there were progressive reductions from approximately 90% to 60% (PLK strain) and to 71.7% (RH strain) in the viable rates of parasites for the IC condition over the incubation period. The parasite viability levels maintained at RT dropped to 73.6% and 50.9% for PLK strain; and 85.2% and 71.7% for RH strain at the 3^rd^ and 6^th^ hpi, respectively (Figures-2c and 3c). Similarly, there were continuous reductions of approximately 30% and 20% in the viability of tachyzoites in MEM at IC, whereas for the RT conditions, there were gradual declines from more than 90% to 55.3% and 64.3% in the viable rates of PLK and RH strains, respectively (Figures-2d and 3d).

One-way repeated ANOVA and the Friedman test showed that the levels of viable parasites were significantly different at different times (p<0.05 as shown in every chart).

## Discussion

Several factors, such as temperature, media, pH, and time, may impact the survival of *T. gondii* tachyzoites [9,13,18]. The present result showed that NS was the best medium for short-term storage (within 6 h) of *T. gondii* tachyzoites without 5% CO_2_ supplementation. The viability of the parasites in this solution was significantly higher than in the other media made from MEM comprising many ingredients. NS is preferred as an isotonic solution, widely used in medical and pediatric practice, and in scientific research, especially for animal inoculation [19,20]. The results were comparable to other studies in which nutrient-free media, such as NS and PBS, were more suitable than minimum essential media for maintaining *T. gondii* tachyzoites. Indeed, the media without nutrients, vitamins, carbohydrates, and fats could prolong the viability of free *T. gondii* tachyzoites better than those containing these kinds of supplements [12,13]. The osmotic coefficient of NS is about 0.93 [21] and is similar to the osmolarity of blood in which circulating tachyzoites have been found in a range of host species [22-26]. However, NS is usually an acidic solution with a pH of about 5.5 that might be potentially harmful to the *T. gondii* tachyzoites [14,27]. Indeed, *T. gondii* tissue cysts have a high pH tolerance, but the tachyzoites were extremely sensitive to a solution with a pH of less than 6.0 [14,28]. Therefore, a fresh medium with an adjusted pH 7.4 is vital for the short-term storage of tachyzoites because the pH easily falls when NS absorbs carbon dioxide in the atmosphere [27].

The second highest mean survival was found in the parasites incubated in PBS, except for the RH strain at RT. Interestingly, in contrast with the subtle variations in parasite survival in NS over the preservation period at both temperatures, PBS supported tachyzoite viability for only a short time, particularly at RT. Hence, the inoculation of tachyzoites maintained in PBS should be conducted within 3 h at IC or 1 h at RT after harvesting the parasites.

The *T. gondii* tachyzoites can invade any nucleated cells in the host or cell media and reside in the parasitophorous vacuole membrane (PVM). This membrane is host-derived yet extensively modified by parasites to propagate nutrient acquisition and avoid host immunity [29]. Tachyzoites replicate exclusively inside the PV and then lyse the host cell before the next round, increasing their number and extent of intracellular life [30,31]. PVM is freely permeable to vitamins, sugars, amino acids, nucleobases, nucleosides, and nucleotides that support parasite proliferation [32]. These small nutrients are transported passively from extracellular to inside the cell via the PVM pores constituted by parasite proteins, including GRA17 and GRA24 [33-35]. In addition, the uptake of proteins and lipids is involved in an intravacuolar membranous tubule-vesicular network consisting of membranous tubules and vesicles connecting the PVM and the parasites [36,37].

Contrastly, it was suggested that the resistance of extracellular tachyzoites is quite restricted in cell-free media and that the live tachyzoites could be undetected after 24 h of incubation; furthermore, they cannot proliferate without host cells [10,13,38]. However, free-living protozoans have demonstrated a higher number of computationally annotated transporter families in the genomes than the parasitic organisms [39], greatly stimulating the metabolism of these extracellular parasites. Clearly, the more nutrients they obtain from the media, the more metabolic materials they excrete. It was likely that these biological activities produce harmful materials and cause changes in solution pH, threatening the parasite’s survival. In addition, it showed that tachyzoites could survive longer at 4°C than at 18-22°C and 37°C because they retain a minimum metabolic rate [12,13]. Therefore, enrichment of the nutrient solution, such as with the MEM or MEM-FBS media, and at RT might be more harmful to free-living tachyzoites than NS and PBS for the IC condition.

In addition, the MEM used in this experiment comprised both L-glutamine and Earle’s salts, which are crucial for mammalian cell cultivation. However, they somehow might impact negatively on cells in the closed system. First, L-glutamine is an amino acid serving as an auxiliary energy source for protein and nucleic acid syntheses when the cells rapidly multiply. Nonetheless, L-glutamine is not stable in solution, unlike most other amino acids. Glutamine degradation results in the equimolar formation of ammonia, which is toxic to cells [37,38]. In fact, the rate at which degradation proceeds depends on time, temperature, type, and the pH of the buffer. Degradation of 0.23%/day occurred in water at pH 6.5 and 0.8%/day in mixed total parenteral nutrition solution at 22-24°C. The L-glutamine degradation rate in the intravenous solutions was <0.15%/day at 4°C [40,41]. Therefore, the parasites incubated in MEM and MEM-FBS with IC conditions had higher survival proportions than at RT in this study.

Another important point is that Earle’s salts have a high sodium bicarbonate level, so they should be used in a 5% CO_2_ air environment. Because MEM supplemented with these salts was available for the cell cultivation system in the laboratory, it was compared with other solutions in this study. However, MEM with Hank’s salts having a lower sodium bicarbonate level has been recommended in the closed system without 5% CO_2_ to enhance the viability of tachyzoites [42].

The viability of tachyzoites in MEM-FBS was relatively higher than for MEM, which had the lowest proportion in almost all groups. The result agreed with other studies that reported that the addition of serum helped prolong free, living tachyzoites [9,10,13]. BSA, a principal component of FBS, is considered a microcarrier, making macromolecules more available for tachyzoites; thus, it could increase parasite survival [43].

## Conclusion

NS was preferable to the other media for short-term storage of free, purified *T. gondii* tachyzoites for IC and RT conditions. Using this preservative with the IC condition is likely to help prolong the viability of *T. gondii* tachyzoites by several hours without a 5% CO_2_ supplementation, which may benefit long-distance transportation. Because extracellular tachyzoites are extremely sensitive to the acidic media, the pH of the used media should be measured at the end of the incubation period to observe its change. This was a limitation in this study because of the small volume of residual media. Trypan blue exclusion is a simple and rapid technique to evaluate the viability of parasites. Yet, the viable tachyzoites are assessed subjectively, and small amounts of dye uptake indicating cell injury may go undetected. Thus, the infectivity of parasitic *T. gondii* tachyzoites might be needed to confirm with *in vivo* assay in animals.

## Authors’ Contributions

TI and KK: Planned and designed the study and revised the manuscript. WP: Analyzed the data. NTT: Conducted the study, analyzed the data, and drafted the manuscript. All authors have read and approved the final manuscript.
